# Meta-Analysis and Bioinformatics Detection of Susceptibility Genes in Diabetic Nephropathy

**DOI:** 10.3390/ijms23010020

**Published:** 2021-12-21

**Authors:** Maria Tziastoudi, Christos Cholevas, Theoharis C. Theoharides, Ioannis Stefanidis

**Affiliations:** 1 Department of Nephrology, Faculty of Medicine, School of Health Sciences, University of Thessaly, 41500 Larisa, Greece; stefanid@med.uth.gr; 2 First Department of Ophthalmology, Faculty of Health Sciences, Aristotle University of Thessaloniki School of Medicine, AHEPA Hospital, 54636 Thessaloniki, Greece; ccholevas@auth.gr; 3 Department of Immunology, Tufts University School of Medicine, Boston, MA 02111, USA; theoharis.theoharides@tufts.edu

**Keywords:** diabetic nephropathy, meta-analysis, gene ontology, protein network, genes, signaling pathway

## Abstract

The latest meta-analysis of genome-wide linkage studies (GWLS) identified nine cytogenetic locations suggestive of a linkage with diabetic nephropathy (DN) due to type 1 diabetes mellitus (T1DM) and seven locations due to type 2 diabetes mellitus (T2DM). In order to gain biological insight about the functional role of the genes located in these regions and to prioritize the most significant genetic loci for further research, we conducted a gene ontology analysis with an over representation test for the functional annotation of the protein coding genes. Protein analysis through evolutionary relationships (PANTHER) version 16.0 software and Cytoscape with the relevant plugins were used for the gene ontology analysis, and the overrepresentation test and STRING database were used for the construction of the protein network. The findings of the over-representation test highlight the contribution of immune related molecules like immunoglobulins, cytokines, and chemokines with regard to the most overrepresented protein classes, whereas the most enriched signaling pathways include the VEGF signaling pathway, the Cadherin pathway, the Wnt pathway, the angiogenesis pathway, the p38 MAPK pathway, and the EGF receptor signaling pathway. The common section of T1DM and T2DM results include the significant over representation of immune related molecules, and the Cadherin and Wnt signaling pathways that could constitute potential therapeutic targets for the treatment of DN, irrespective of the type of diabetes.

## 1. Introduction

The genetic background of diabetic nephropathy (DN) remains obscure, although unquestionable [1,2,3]. Many loci have been implicated in the genetic architecture of DN through different methodological approaches [4,5,6]. Genetic linkage studies and genetic association studies are the two main approaches used in the genetic dissection of DN and other complex traits [7,8].

Three genome wide linkage studies (GWLS) were conducted in probands with DN due to T1DM [9,10,11]. The primary findings of Rogus et al. was on chromosome 19q, with a secondary peak on chromosome 2q [11]. A novel locus at cytogenetic location 22q11 was identified as significant of linkage with DN. In addition, this study confirms the importance of previously indicated DN loci on 3q21–q25 and 19q13 [9]. Finally, Österholm et al. suggest that the locus on 3q harbors a susceptibility gene for DN [10].

Several GWLS have also been conducted for the genetic dissection of DN due to T2DM. Strong and suggestive evidence for a linkage with DN was detected in 6p and 7p, respectively [12]. Bowden et al. (2004) concluded that maybe a susceptibility locus for DN due to T2DM is located on chromosome 7p [13]. Other regions suggestive of a linkage with DN secondary to T2DM are the chromosomal regions 3q and 18q [13]. Lastly, one more study detected strong evidence for linkage to DN at 7q, and suggestive evidence at 3q, 9q, and 20p [14].

However, the findings of the individual genetic linkage studies have been inconclusive. In an effort to produce more consistent evidence, a meta-analysis of GWLS was performed [15]. With regard to DN due to T1DM, the meta-analysis identified nine regions as suggestive of linkage to DN, which are located on seven chromosomes [15]. Regarding DN due to T2DM, the meta-analysis revealed significance of linkage to DN at seven regions that are located on five different chromosomes.

GWLS often produce many cytogenetic locations as suggestive for linkage with a trait or a disease harboring hundreds or even thousands of genetic loci. The next step is the prioritization of the most promising genes for biological validation [16,17]. Because proteins do not act individually, it is extremely informative to explore protein−protein interactions (PPIs) in complex protein networks. STRING is a web tool that provides a network view of a set of proteins and is very useful when one seeks insight into the biological functions and signaling pathways that might be involved in the pathogenesis of a disease, because it provides the gene ontology (GO) analysis of the inputted set of proteins and also the most deregulated signaling pathways of KEGG and/or the Reactome database [18,19,20].

In order to prioritize the most relevant genes among the genes harbored in specific locations identified by a meta-analysis of GWLS, we performed both gene ontology and enrichment analysis, as well as protein network analysis, for the identification of the key genes in DN secondary to T1DM or T2DM exclusively.

## 2. Results

**Figure 1:**
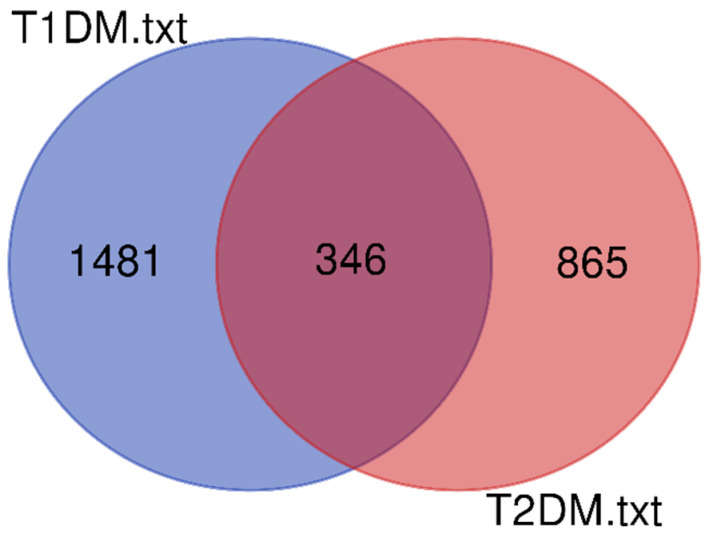
Venn diagram regarding the protein coding genes in T1DM and T2DM.

In the nine cytogenetic locations that were revealed to be significant in the T1DM subgroup meta-analysis of GWLS (1q43–1q44, 3q21.2–3q25.32, 5q11.2–5q14.3, 5q14.3–5q23.2, 16p12.3–16q12.2, 17q24.3–17q25.3, 19q13.33–19q13.43, 22p13–22q12.3, and 22q12.3–22q13.3), 3500 genetic loci were harbored, out of which 1827 loci were protein coding genes (Table 1). In the seven cytogenetic locations that were revealed to be significant in the T2DM subgroup meta-analysis of GWLS (4p14–4q13.3, 5q14.3–5q23.2, 5q23.2–5q34, 7p22.3–7p15.3, 7q22.3–7q34, 15q11.2–15p13, and 22p13–22q12.3), 2619 genetic loci were included, out of which 1211 protein coding genes were located. The common section of the protein coding genes in T1DM and T2DM constituted 346 genes (Figure 1, Table S1).

### 2.1. GO Analysis and over Representation Test

#### 2.1.1. GO Analysis Results

In T1DM and regarding the principal molecular functions of the genes, most of the genes are involved in the binding process, catalytic activity, molecular function regulation, and transporter and molecular transducer activity. With regard to the principal biological processes, most of the genes are implicated in a cellular and metabolic process, in biological regulation, in response to stimulus, and in localization. Regarding the principal cellular components, most gene products are localized in a cellular anatomical entity, intracellularly, and in a protein-containing complex. The five protein classes with the most genes are a metabolite interconversion enzyme, a protein modifying enzyme, a gene-specific transcriptional regulator, a transmembrane signal receptor, and a nucleic acid metabolism protein. The majority of the genes are involved in certain signaling pathways, like angiogenesis, integrin signaling pathway, inflammation mediated by chemokine and cytokine signaling pathway, Gonadotropin-releasing hormone receptor pathway, and EGF receptor signaling pathway (Table 2) (Figures S1–S5).

In T2DM, the principal molecular functions, biological processes, and cellular components of the genes are identical to these of T1DM. Regarding the five protein classes with the most genes, these are a metabolite interconversion enzyme, a protein modifying enzyme, transporters, cell adhesion molecules, and gene-specific transcriptional regulators. The signaling pathways with the most of the genes involved are the Wnt signaling pathway, cadherin signaling pathway, EGF receptor signaling pathway, gonadotropin-releasing hormone receptor pathway, and angiogenesis (Table 2) (Figures S6–S10).

#### 2.1.2. Over Representation Test Results

An overrepresentation test with PANTHER v16.0 followed the GO analysis. In T1DM, the top five most enriched biological processes are heterocycle biosynthetic process, biosynthetic process, aromatic compound biosynthetic process, organic substance biosynthetic process, and transcription by RNA polymerase II. The top five molecular functions are the RNA polymerase II cis-regulatory region sequence-specific DNA binding and, more specifically, the cis-regulatory region sequence-specific DNA binding, the transcription regulatory region sequence-specific DNA binding, the regulatory region nucleic acid binding, and the nucleic acid binding. Regarding the most enriched cellular components, these include a secretory granule, a secretory vesicle, a membrane-bounded organelle, an organelle, and an intracellular organelle. With regard to the top five statistically significant enriched protein classes, these are the immunoglobulin superfamily cell adhesion molecule, the immunoglobulin receptor superfamily, the C2H2 zinc finger transcription factor, the zinc finger transcription factor, and the serine protease. Regarding the most enriched signaling pathways, the VEGF signaling pathway, the Cadherin signaling pathway, the angiogenesis pathway, the p38 MAPK pathway, and the EGF receptor signaling pathway (Table 3).

In T2DM, the most enriched biological processes include biological adhesion, cell adhesion, peptidyl-tyrosine modification, cellular response to stimulus, and response to a molecule of bacterial origin. The most overrepresented molecular functions include the chloride transmembrane transporter activity and, more specifically, inorganic anion transmembrane transporter activity and anion transmembrane transporter activity, while the most enriched cellular components include integral and intrinsic components of the plasma membrane. The most overrepresented protein classes include cadherins, cell adhesion molecules, chemokines, cytokines, and intercellular signal molecules. Lastly, the most enriched pathways include those of the Cadherin and Wnt signaling pathways (Table 4).

### 2.2. Protein Network Analysis

**Figure 2:**
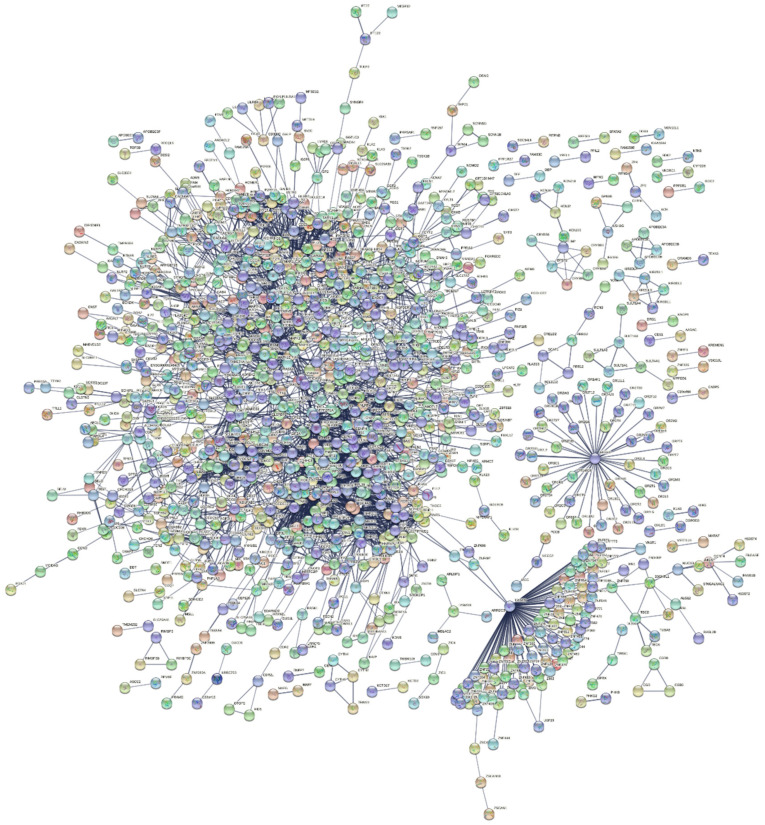
STRING protein network with 1779 nodes and 3761 edges (the edges indicate both functional and physical protein associations) regarding T1DM.

Regarding T1DM, in order to further elucidate the function of the 1827 genes, we constructed a PPI network that includes 1779 nodes and 3761 edges, with an average node degree of 4.23 by using the STRING database and Cytoscape software (Figure 2). The PPI enrichment *p*-value was 6.78 × 10^−12^, which means that the network had significantly more interactions than expected.

**Figure 3:**
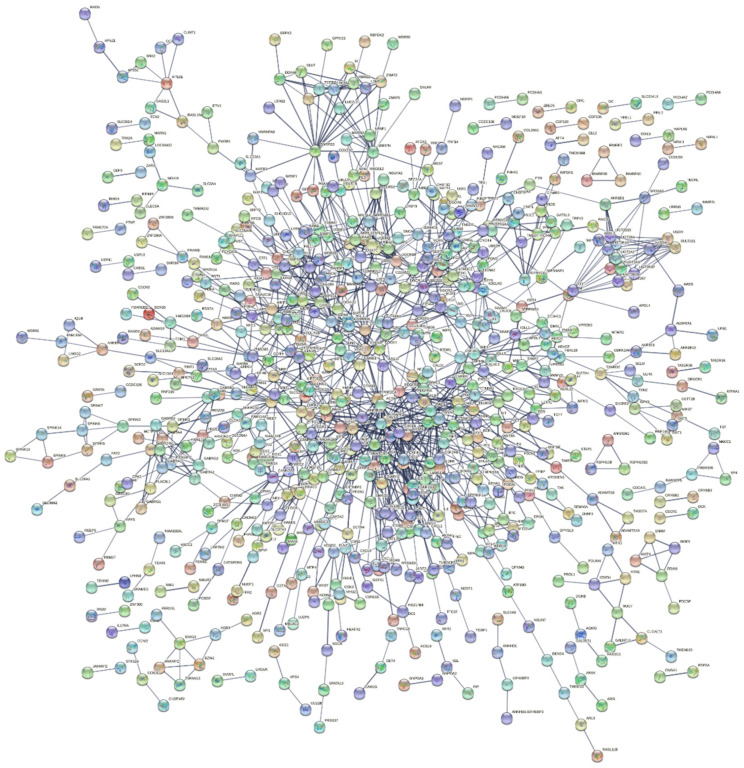
STRING protein network with 1197 nodes and 1313 edges (the edges indicate both functional and physical protein associations) regarding T2DM.

Regarding T2DM, in order to elucidate further the function of the 1211 genes, we constructed a PPI network that included 1197 nodes and 1313 edges, with an average node degree of 2.19, by using the STRING database and Cytoscape software (Figure 3). The PPI enrichment *p*-value was <1.0 × 10^−16^, which means that the network had significantly more interactions than expected.

**Figure 4:**
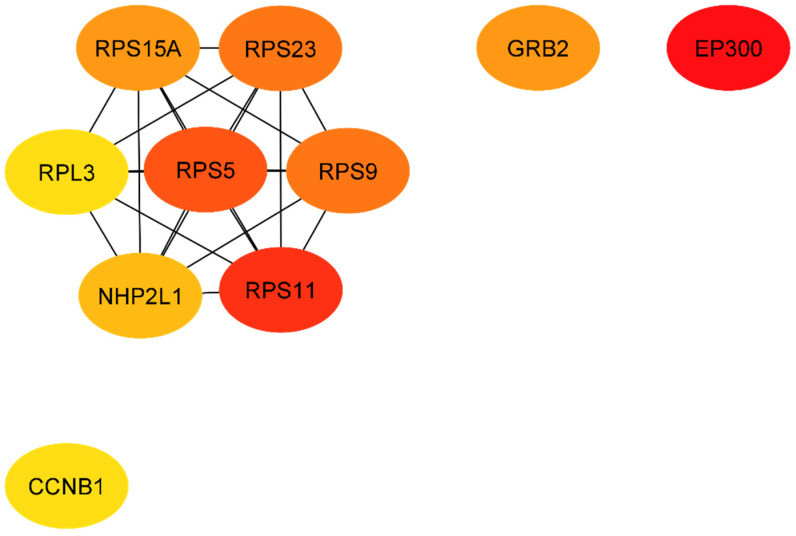
The top 10 nodes based on their degree in T1DM protein network.

**Figure 5:**
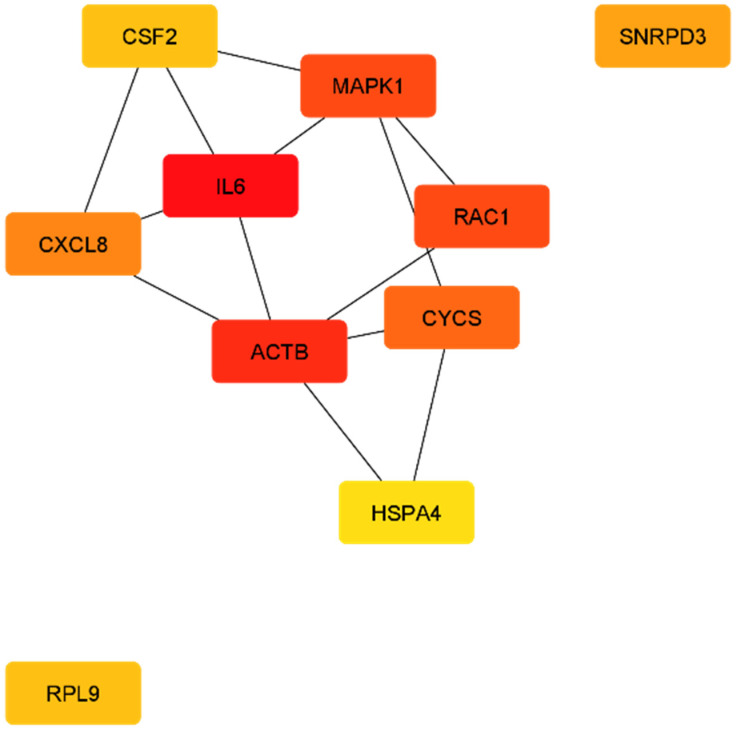
The top 10 nodes based on their degree in T2DM protein network.

The protein network analysis by the CytoHubba plugin revealed the following 10 genes as the nodes with the most interactions in T1DM-DN: EP300, RPS11, RPS5, RPS23, RPS9, GRB2, RPS15A, NHP2L1, CCNB1, and RPL3 (Table 5 and Figure 4). In T2DM, the 10 genes with the most interactions in the protein network are: *IL6, ACTB, MAPK1, RAC1, CYCS, CXCL8 (IL8), SNRPD3, CSF2, RPL9,* and *HSPA4* (Table 5) (Figure 5). In Table S2, we report the next top 10 genes per diabetes type.

## 3. Discussion

With the current bioinformatics study, we identified the functional role of the protein coding genes located in the cytogenetic locations that were statistically significant in a meta-analysis of genome-wide linkage studies in T1DM or T2DM exclusively, in an effort to detect these dysregulated pathways and the key genes that are responsible in each type of diabetes [15]. For this purpose, we conducted a genetic ontology analysis and over-representation test with PANTHER v16.0 in order to highlight the most over-represented GO terms of these genes, and analyzed the protein network of these genes in order to find the hub genes with STRING and Cytoscape apps.

Among the most over-represented protein classes of the protein coding genes in both T1DM and T2DM analyses are the molecules related to immune responses. More specifically, in the T1DM analysis, some of the most enriched protein classes include the immunoglobulin superfamily cell adhesion molecule and immunoglobulin receptor superfamily, while in T2DM, chemokines and cytokines are among the most enriched protein classes. Although DN is not a classical immune disease, the crucial role of the immune system contribution in the course of the disease is undoubtedly present. More specifically, immunoglobulin G (IgG), immunoglobulin M (IgM), and complement deposition are common findings in the kidneys of patients with DN, as they have been depicted by immunofluorescence staining [21]. It has been also found that the deposition of glomerular C4c is a predictor of an unfavorable renal outcome [22]. A meta-analysis of the genetic association studies regarding patients with DN revealed the significance of variants in *CCL2*, *CCR5*, *IL6*, *IL8*, *EPO*, *IL1A*, *IL1B*, *IL100*, *IL1RN*, *GHRL*, *MMP9*, *TGFB1*, *VEGFA*, *MMP3*, *MMP12*, *IL12RB1*, *PRKCE*, *TNF,* and *TNFRSF19* genes in the development of DN [5]. Levels of several cytokines, such as IL-6, IL-18, and TNF, are elevated in patients with DN [23,24,25]. Proteinuria, a hallmark symptom of DN, is not only evidence of the underlying renal damage, but also a cause for further injury and immune response to the underlying tissue injury.

In addition, one of the most over-represented pathways is angiogenesis. Indeed, experimental data indicate that VEGF is responsible for the increase of neovascularization observed in patients with DN, which is correlated with the expression of VEGF and angiopoietin [26]. VEGF is expressed mainly in podocytes, distal tubules, and collecting ducts, while its receptors are harbored on glomerular endothelial cells [27]. Although hypoxia is the main stimulatory factor of VEGF, several cytokines, growth factors, and other factors, out of which many are related to DN such as hyperglycemia, advanced glycation end products (AGEs), prostaglandins, mechanical stress, protein kinase C (PKC), reactive oxygen species (ROS), angiotensin II, and aldose reductase, also induce VEGF [27,28]. Renal expression of VEGF and its receptors is up-regulated in patients of both type 1 and type 2 diabetes, especially early in the onset of diabetes, whereas the inhibition of VEGF with anti-VEGF-antibodies acts beneficially in the renal changes induced by diabetes, indicating a harmful role of this factor in the pathophysiology of DN [27,29]. VEGFA G405C, -1499C > T, and -2549 I/D are some of the genetic variants examined in genetic association studies in patients with DN due to either T1DM or T2DM [30,31,32,33,34]. Nikzamir et al. (2012) identified a statistically significant association between VEGF +405 GG variant and DN [28]. VEGF-2549 D/I polymorphism, and more specifically the D allele, was associated with susceptibility to DN in type 1 diabetics [35]. VEGFA is also implicated, except in DN, in polycystic kidney disease, suggesting that its role is significant for the maintenance of good functioning of the renal vascular system [36,37].

One of the most significantly enriched signaling pathways either in T1DM or T2DM is also the Cadherin pathway. This finding confirms the results of our previous in silico study, which is referred to DN secondary to both types of diabetes, T1DM and T2DM, and demonstrated the contribution of Wnt and Cadherin signaling pathways in the pathogenesis of DN [38]. Previous data indicate that an altered cadherin expression is an early biomarker of DN, as its levels are elevated early in the course of the disease and continue to elevate during disease progression [39,40]. Dysregulated cadherin expression is also implicated in DN associated proteinuria because it is involved in epithelial−mesenchymal transition (EMT) [41]. The fact that this pathway is enriched in all types of analysis indicates the potential crucial role of it, and it could possibly serve as a therapeutic target as it seems to be related with diabetic nephropathy per se, irrespective of the type of the underlying diabetes.

One more finding of the present bioinformatics study with regard to T2DM analysis confirms the results of our previous in silico analysis about the significant enrichment of the Wnt signaling pathway [38]. Previous data indicate the convergence between Wnt, β-catenin, and cadherin pathways [42]. Wnts are growth factor signaling molecules that are involved in many biological processes, such as cell−cell adhesion, proliferation, differentiation, and cell migration [42]. Upregulation of the Wnt/β-catenin signaling pathway dysregulated podocyte function in DN [43,44,45]. Other lines of evidence come from studies in several diabetic experimental animals in which the levels of Wnt proteins were elevated compared to the non diabetic controls [46]. The role of Wnt pathway is more extensively discussed in our previous analysis [38].

The EGF receptor signaling pathway is also one of the most enriched pathways in DN due to T1DM. EGF is the most well studied ligand of EGFR which is expressed throughout the kidney epithelium and interstitium [47]. EGF could serve as a prognostic biomarker of kidney diseases because it is excreted partially in the urine [48]. It is noteworthy to mention that EGF levels increase in the serum of patients with DN at all stages of chronic kidney disease (CKD) compared to healthy controls [49]. More evidence about the implication of EGFR signaling in DN is the fact that the inhibition of EGFR preserved the podocytes number and reduced albuminuria [50]. Therefore, the EGFR pathway could function as a potential therapeutic target. The EGFR pathway also has a crucial role in nephrogenesis, while in adults, this pathway controls the electrolyte handling and more specifically in sodium, calcium, and magnesium homeostasis [51]. EGFR pathway activation leads to activation of the mitogen-activated protein kinase (MAPK) and phosphatidylinositol 3-kinase (PI3K) signaling pathways [51]. In addition, the expression of p38 MAPK and Wnt/β-catenin were significantly increased in an animal model of cadmium-induced diabetic nephropathy [52].

One new or unconventional pathway linked to T1DM-DN or T2DM-DN was the gonadotropin-releasing hormone receptor pathway. However, after extensive literature research, we found that this pathway controls miRNAs that are upregulated after hemodialysis. Therefore, the authors concluded that hemodialysis alters the circulating miRNAs and this alteration affects gonadotropin-releasing hormone receptor, cell cycle, and cell pluripotency-related pathways, which are associated with subfertility and increased risk for cancer development, conditions that have been associated with hemodialysis [53].

Novel hub genes, genes with the most interactions in the protein networks, we noticed in both T1DM-DN and T2DM-DN protein networks. In the case of T1DM, all the hub genes were novel findings. We searched in the literature for genetic association studies regarding these genes, but we did not find any studies. On the other hand, in the T2DM-DN protein network, the hub genes include two genes already examined in the context of genetic association studies, *IL6* and *CXCL8*. It is interesting that many of the hub genes are harbored in chromosome 5q, which was one of the common significant regions in both T1DM and T2DM in the meta-analysis whose data we used in the present study.

It is noteworthy to mention that MODY and ketosis-prone T2DM were not included in this analysis because there was not a sufficient number of linkage studies regarding MODY in order to be included in the meta-analysis. Future studies should also investigate the role of the underlying genetics of these types of diabetes, although the available studies are less common in the literature than the studies regarding the T1DM and T2DM, and thus it is not likely to change the results of the current study.

Last, but not least, the importance and the novelty of this study regards the methodological approach used, because it offers a new approach in the interpretation of the results of genetic linkage studies. This approach is more common in the case of the results derived from genetic association studies. In the context of the present study, we extended the use of genetic ontology analysis and protein network analysis in the results of linkage studies of diabetic nephropathy. It is interesting that we replicated the results from other methodological approaches. This convergence strengthens our results and constitutes further evidence for the credibility of our conclusions.

## 4. Materials and Methods

### 4.1. Data Sources

The data are derived from the most recent meta-analysis of GWLS in DN [15]. DN was defined in the presence of persistent micro-/macro-albuminuria and/or chronic renal insufficiency, whereas other causes of nephropathy, except longstanding T1DM and T2DM were excluded. GWLS of albuminuria, estimated glomerular filtration rate (eGFR) and serum creatinine were excluded from the meta-analysis [15]. In order to find which genes are located in the significantly suggestive regions for linkage with DN, we used the University of California Santa Cruz (UCSC) Genome Browser (https://genome.ucsc.edu/, accessed on 13 October 2021) and, more particularly, the assembly December 2013 (GRCh38/hg38) [54]. The aforementioned meta-analysis regarding only the T1DM sub-analysis identified nine cytogenetic locations (1q43–1q44, 3q21.2–3q25.32, 5q11.2–5q14.3, 5q14.3–5q23.2, 16p12.3–16q12.2, 17q24.3–17q25.3, 19q13.33–19q13.43, 22p13–22q12.3, and 22q12.3–22q13.3) as significantly suggestive for linkage with DN in T1DM, and seven regions (4p14–4q13.3, 5q14.3–5q23.2, 5q23.2–5q34, 7p22.3–7p15.3, 7q22.3–7q34, 15q11.2–15p13, and 22p13–22q12.3) as significantly suggestive for linkage with DN in T2DM [15].

### 4.2. GO Analysis and Statistical Significance

In order to delineate the biological role of the genes harbored in the locations identified from the meta-analysis, we focused on the role of the protein coding genes. For this purpose, the PANTHER functional classification system was used [55,56]. Statistical overrepresentation test with PANTHER was performed after the GO analysis. Fisher’s exact test was used for the calculation of the statistical significance and was adjusted using the false discovery rate (FDR) for the correction of multiple tests. An FDR-corrected *p* value threshold of <0.05 was used.

### 4.3. PPI Network Construction and Selection of Hub Genes

The STRING (Search Tool for the Retrieval of INteracting Genes) (https://string-db.org, accessed on 13 October 2021) database was used to construct the protein network for the protein coding genes located in the locations identified from the meta-analysis of GWLS [15]. The minimum confidence score was set at 0.700. Cytoscape (version 3.8.2, https://cytoscape.org/index.html, accessed on 13 October 2021) was used for PPI network visualization [57]. The CytoHubba app in Cytoscape was used to detect the 10 hub genes with the highest scores [53].

## 5. Conclusions

Taken together, in the present study, we analyzed in silico the findings of a meta-analysis of genome-wide linkage studies and prioritized the most probable candidate genes involved in DN in the context of T1DM or T2DM. The significance of our findings rests on the identification of immune-related molecules, as well as the importance of Cadherin and Wnt signaling pathways. Our findings may indicate potential biomarkers or therapeutic targets for DN per se, irrespective of the type of underlying diabetes.

## Figures and Tables

**Table 1:** Identification of gene type.

Gene Type	T1DM (# Genes)	T2DM (# Genes)
protein coding	1827	1211
pseudo	1004	818
ncRNA	584	363
snRNA	3	2
snoRNA	31	104
rRNA	−	1
other	51	120
Total	3500	2619

**Table 2:** The top five GO terms per type of diabetes.

GO Term	T1DM-DN		T2DM-DN	
GO Term	Top 5 GO Terms	# of Genes	Top 5 GO Terms	# of Genes
Molecular Function				
	binding (GO:0005488)	592	binding (GO:0005488)	330
	catalytic activity (GO:0003824)	378	catalytic activity (GO:0003824)	242
	molecular function regulator (GO:0098772)	281	molecular function regulator (GO:0098772)	114
	transporter activity (GO:0005215)	79	transporter activity (GO:0005215)	65
	molecular transducer activity (GO:0060089)	54	molecular transducer activity (GO:0060089)	53
Biological Process				
	cellular process (GO:0009987)	938	cellular process (GO:0009987)	605
	metabolic process (GO:0008152)	621	metabolic process (GO:0008152)	343
	biological regulation (GO:0065007)	567	biological regulation (GO:0065007)	313
	response to stimulus (GO:0050896)	240	response to stimulus (GO:0050896)	169
	localization (GO:0051179)	212	localization (GO:0051179)	165
Cellular Component				
	cellular anatomical entity (GO:0110165)	1035	cellular anatomical entity (GO:0110165)	720
	intracellular (GO:0005622)	822	intracellular (GO:0005622)	484
	protein-containing complex (GO:0032991)	249	protein-containing complex (GO:0032991)	171
Protein Class				
	metabolite interconversion enzyme (PC00262)	197	metabolite interconversion enzyme (PC00262)	115
	protein modifying enzyme (PC00260)	134	protein modifying enzyme (PC00260)	83
	gene-specific transcriptional regulator (PC00264)	133	transporter (PC00227)	74
	transmembrane signal receptor (PC00197)	103	cell adhesion molecule (PC00069)	62
	nucleic acid metabolism protein (PC00171)	98	gene-specific transcriptional regulator (PC00264)	55
Pathway				
	Angiogenesis (P00005)	26	Wnt signaling pathway (P00057)	71
	Integrin signalling pathway (P00034)	24	Cadherin signaling pathway (P00012)	62
	Inflammation mediated by chemokine and cytokine signaling pathway (P00031)	24	EGF receptor signaling pathway (P00018)	15
	Gonadotropin-releasing hormone receptor pathway (P06664)	20	Gonadotropin-releasing hormone receptor pathway (P06664)	14
	EGF receptor signaling pathway (P00018)	20	Angiogenesis (P00005)	14

**Table 3:** The top five results of the over-representation test in T1DM-DN.

	Homo Sapiens (REF)	Client Text Box Input (Hierarchy )					
**PANTHER GO-Slim Biological Process**	**#**	**#**	**Expected**	**Fold Enrichment**	**+/−**	**Raw *p* Value**	**FDR**
heterocycle biosynthetic process	2322	277	208.24	1.33	+	3.52 × 10^−6^	1.10 × 10^−3^
biosynthetic process	3011	346	270.03	1.28	+	4.13 × 10^−6^	1.13 × 10^−3^
aromatic compound biosynthetic process	2323	276	208.33	1.32	+	5.06 × 10^−6^	1.23 × 10^−3^
organic substance biosynthetic process	3004	346	269.40	1.28	+	3.44 × 10^−6^	1.25 × 10^−3^
transcription by RNA polymerase II	1635	205	146.63	1.40	+	5.95 × 10^−6^	1.30 × 10^−3^
**PANTHER GO-Slim Molecular Function**	**#**	**#**	**Expected**	**Fold Enrichment**	**+/−**	**Raw *p* Value**	**FDR**
RNA polymerase II cis-regulatory region sequence-specific DNA binding	1054	162	94.52	1.71	+	7.14 × 10^−10^	3.96 × 1^−7^
cis-regulatory region sequence-specific DNA binding	1057	162	94.79	1.71	+	7.55 × 10^−10^	2.09 × 10^−7^
transcription regulatory region sequence-specific DNA binding	1377	188	123.49	1.52	+	8.28 × 10^−8^	5.11 × 10^−6^
regulatory region nucleic acid binding	1377	188	123.49	1.52	+	8.28 × 10^−8^	4.60 × 10^−6^
nucleic acid binding	2248	274	201.61	1.36	+	8.13 × 10^−7^	3.76 × 10^−5^
**PANTHER GO-Slim Cellular Component**	**#**	**#**	**Expected**	**Fold Enrichment**	**+/−**	**Raw *p* Value**	**FDR**
secretory granule	64	18	5.74	3.14	+	9.85 × 10^−5^	6.25 × 10^−3^
secretory vesicle	146	31	13.09	2.37	+	5.17 × 10^−5^	4.38 × 10^−3^
membrane-bounded organelle	5999	636	538.00	1.18	+	2.42 × 10^−6^	4.09 × 10^−4^
organelle	6781	714	608.13	1.17	+	7.48 × 10^−7^	3.80 × 10^−4^
intracellular organelle	6633	692	594.86	1.16	+	5.06 × 10^−6^	6.43 × 10^−4^
**PANTHER Protein Class**	**#**	**#**	**Expected**	**Fold Enrichment**	**+/−**	**Raw *p* Value**	**FDR**
immunoglobulin superfamily cell adhesion molecule	24	12	2.15	5.58	+	1.80 × 10^−5^	1.16 × 10^−3^
immunoglobulin receptor superfamily	191	36	17.13	2.10	+	1.34 × 10^−4^	6.44 × 10^−3^
C2H2 zinc finger transcription factor	460	84	41.25	2.04	+	2.04 × 10^−8^	3.94 × 10^−6^
zinc finger transcription factor	541	89	48.52	1.83	+	4.55 × 10^−7^	4.39 × 10^−5^
serine protease	198	33	17.76	1.86	+	1.65 × 10^−3^	4.55 × 10^−2^
**PANTHER Pathways**	**#**	**#**	**Expected**	**Fold Enrichment**	**+/−**	**Raw *p* Value**	**FDR**
VEGF signaling pathway	68	14	6.10	2.30	+	7.61 × 10^−3^	1.00
Cadherin signaling pathway	164	5	14.71	.34	−	1.04 × 10^−2^	8.73 × 10^−1^
Angiogenesis	175	26	15.69	1.66	+	1.98 × 10^−2^	1.00
p38 MAPK pathway	41	8	3.68	2.18	+	6.01 × 10^−2^	1.00
EGF receptor signaling pathway	141	20	12.65	1.58	+	6.02 × 10^−2^	1.00

**Table 4:** The top five results of the over-representation test in T2DM-DN.

	Client Text Box Input (Hierarchy)						
**PANTHER GO-Slim Biological Process**	**#**	**#**	**Expected**	**Fold Enrichment**	**+/−**	**Raw *p* Value**	**FDR**
biological adhesion	366	75	21.82	3.44	+	5.83 × 10^−1^	6.36 × 10^−15^
cell adhesion	366	75	21.82	3.44	+	5.83 × 10^−18^	1.27 × 10^−14^
peptidyl-tyrosine modification	53	13	3.16	4.11	+	6.68 × 10^−5^	2.08 × 10^−2^
cellular response to biotic stimulus	41	12	2.44	4.91	+	2.97 × 10^−5^	2.16 × 10^−2^
response to molecule of bacterial origin	45	12	2.68	4.47	+	6.39 × 10^−5^	2.32 × 10^−2^
**PANTHER GO-Slim Molecular Function**	**#**	**#**	**Expected**	**Fold Enrichment**	**+/−**	**Raw *p* Value**	**FDR**
chloride transmembrane transporter activity	79	15	4.71	3.18	+	2.32 × 10^−4^	6.44 × 10^−2^
inorganic anion transmembrane transporter activity	91	17	5.43	3.13	+	1.09 × 10^−4^	6.03 × 10^−2^
anion transmembrane transporter activity	221	29	13.18	2.20	+	2.43 × 10^−4^	4.50 × 10^−2^
**PANTHER GO-Slim Cellular Component**	**#**	**#**	**Expected**	**Fold Enrichment**	**+/−**	**Raw *p* Value**	**FDR**
integral component of plasma membrane	786	110	46.87	2.35	+	1.00 × 10^−14^	2.55 × 10^−12^
intrinsic component of plasma membrane	798	111	47.58	2.33	+	8.84 × 10^−15^	4.49 × 10^−12^
membrane	4165	304	248.34	1.22	+	1.76 × 10^−4^	1.78 × 10^−2^
intrinsic component of membrane	1180	135	70.36	1.92	+	6.44 × 10^−12^	8.18 × 10^−10^
integral component of membrane	1151	133	68.63	1.94	+	5.50 × 10^−12^	9.32 × 10^−10^
**PANTHER Protein Class**	**#**	**#**	**Expected**	**Fold Enrichment**	**+/−**	**Raw *p* Value**	**FDR**
cadherin	113	57	6.74	8.46	+	2.88 × 10^−29^	5.56 × 10^−27^
cell adhesion molecule	203	62	12.10	5.12	+	2.86 × 10^−22^	2.76 × 10^−20^
chemokine	24	11	1.43	7.69	+	2.04 × 10^−6^	1.31 × 10^−4^
cytokine	102	17	6.08	2.80	+	3.59 × 10^−4^	1.39 × 10^−2^
intercellular signal molecule	377	39	22.48	1.73	+	1.72 × 10^−3^	4.75 × 10^−2^
**PANTHER Pathways**	**#**	**#**	**Expected**	**Fold Enrichment**	**+/−**	**Raw *p* Value**	**FDR**
Cadherin signaling pathway	164	62	9.78	6.34	+	2.80 × 10^−26^	4.67 × 10^−24^
Wnt signaling pathway	317	71	18.90	3.76	+	7.53 × 10^−19^	6.29 × 10^−17^

**Table 5:** The 10 nodes with the most edges based on the CytoHubba analysis.

Official Gene Symbol	Official Full Name	Score	Cytogenetic Location
**T1DM-DN**			
*EP300*	E1A binding protein p300	43.0	22q13.2
*RPS11*	ribosomal protein S11	38.0	19q13.33
*RPS5*	ribosomal protein S5	37.0	19q13.43
*RPS23*	ribosomal protein S23	35.0	5q14.2
*RPS9*	ribosomal protein S9	35.0	19q13.42
*GRB2*	growth factor receptor bound protein 2	34.0	17q25.1
*RPS15A*	ribosomal protein S15a	34.0	16p12.3
*NHP2L1* (SNU13)	small nuclear ribonucleoprotein 13	31.0	22q13.2
*CCNB1*	cyclin B1	30.0	5q13.2
*RPL3*	ribosomal protein L3	30.0	22q13.1
**T2DM-DN**			
*IL6*	interleukin 6	30.0	7p15.3
*ACTB*	actin beta	26.0	7p22.1
*MAPK1*	mitogen-activated protein kinase 1	25.0	22q11.22
*RAC1*	Rac family small GTPase 1	25.0	7p22.1
*CYCS*	cytochrome c, somatic	21.0	7p15.3
*CXCL8 (IL8)*	C-X-C motif chemokine ligand 8	20.0	4q13.3
*SNRPD3*	small nuclear ribonucleoprotein D3 polypeptide	19.0	22q11.23
*CSF2*	colony stimulating factor 2	17.0	5q31.1
*RPL9*	ribosomal protein L9	17.0	4p14
*HSPA4*	heat shock protein family A (Hsp70) member 4	16.0	5q31.1

## Supplementary Materials

The following are available online at https://www.mdpi.com/article/10.3390/ijms23010020/s1.
