# Supplementary material for: Meta-Analysis and Bioinformatics Detection of Susceptibility Genes in Diabetic Nephropathy
Source: Int J Mol Sci. 2021 Dec 21;23(1):20. doi: 10.3390/ijms23010020 (PMC8744540; doi:10.3390/ijms23010020)
Supplement: Supplementary file 1 [file ijms-23-00020-s001.zip › Supplementary Figures.pdf]

Supplementary Figures

T1DM

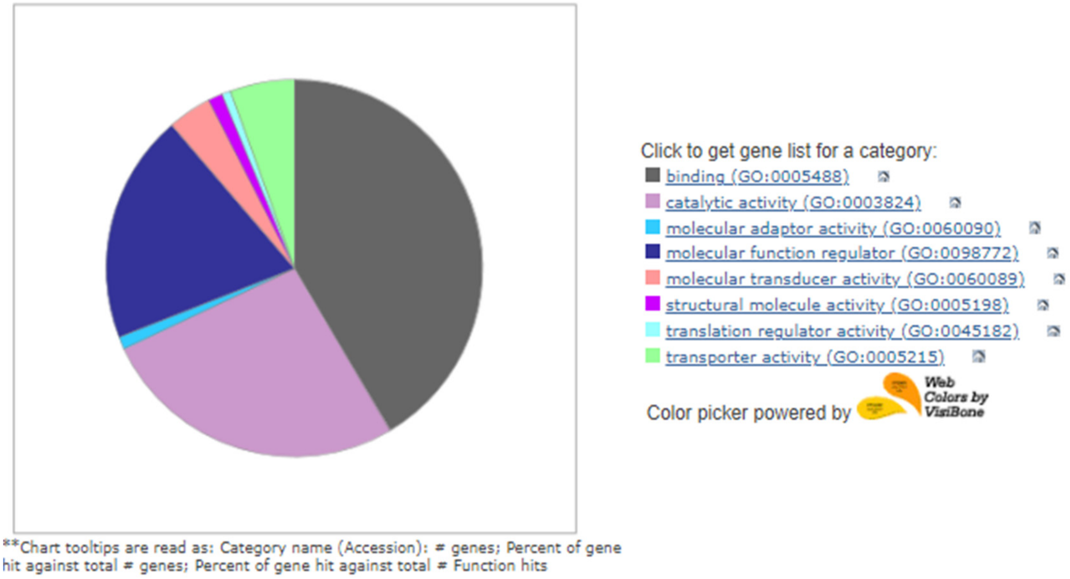

Figure S1: Results of the gene ontology analysis regarding the "molecular function" category.

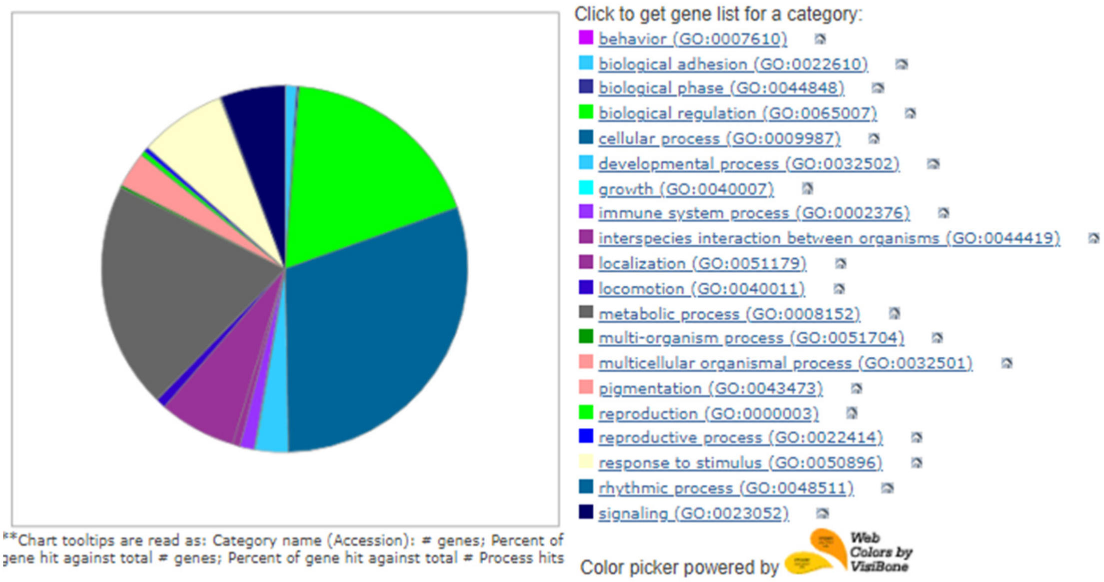

Figure S2: Results of the gene ontology analysis regarding the "biological process" category.

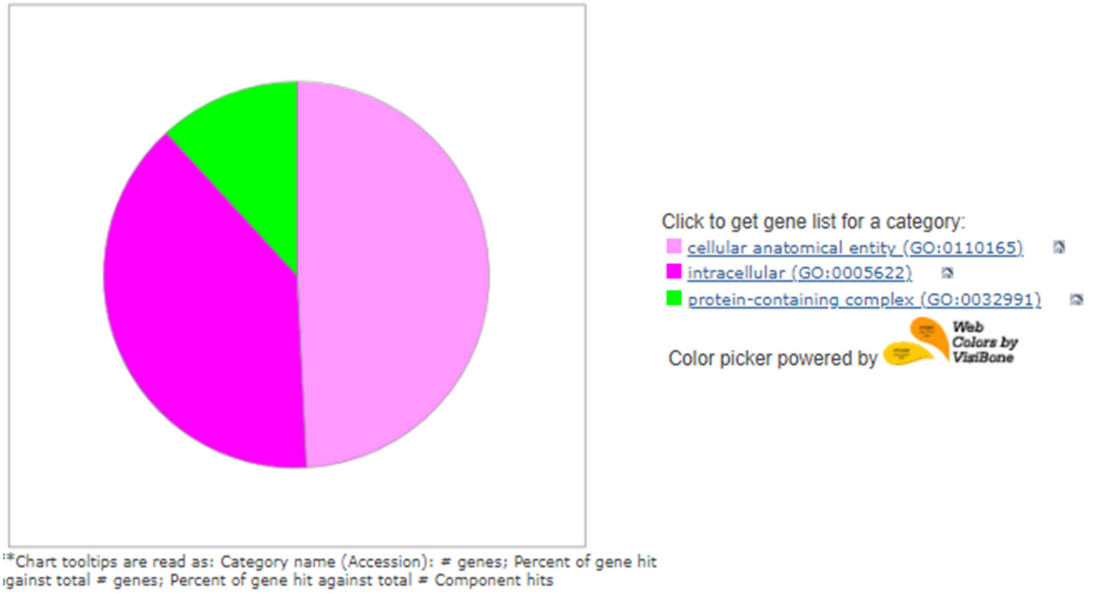

Figure S3: Results of the gene ontology analysis regarding the "cellular component" category.

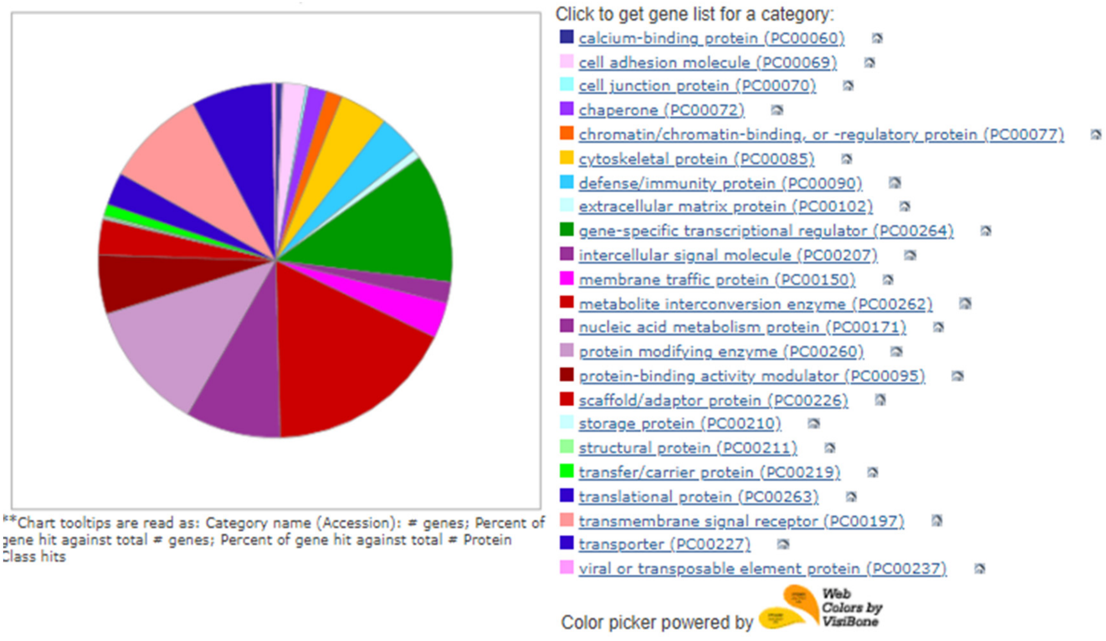

Figure S4: Results of the gene ontology analysis regarding the “protein class” category.

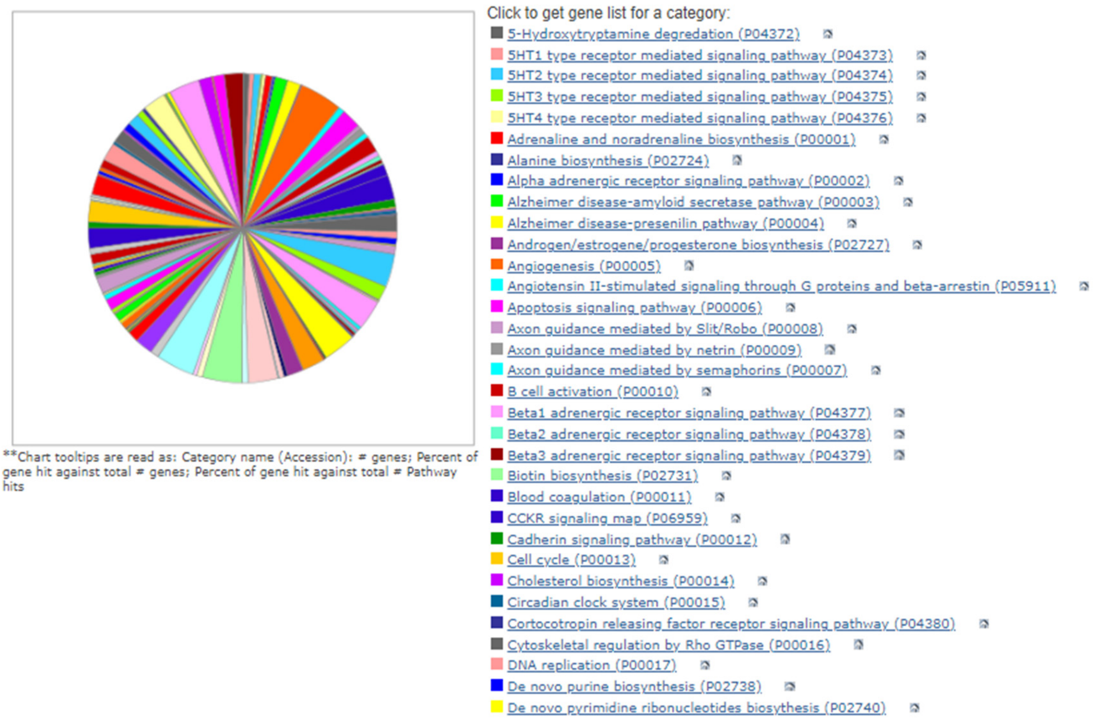

Figure S5: Results of the gene ontology analysis regarding the “pathway” category.

T2DM

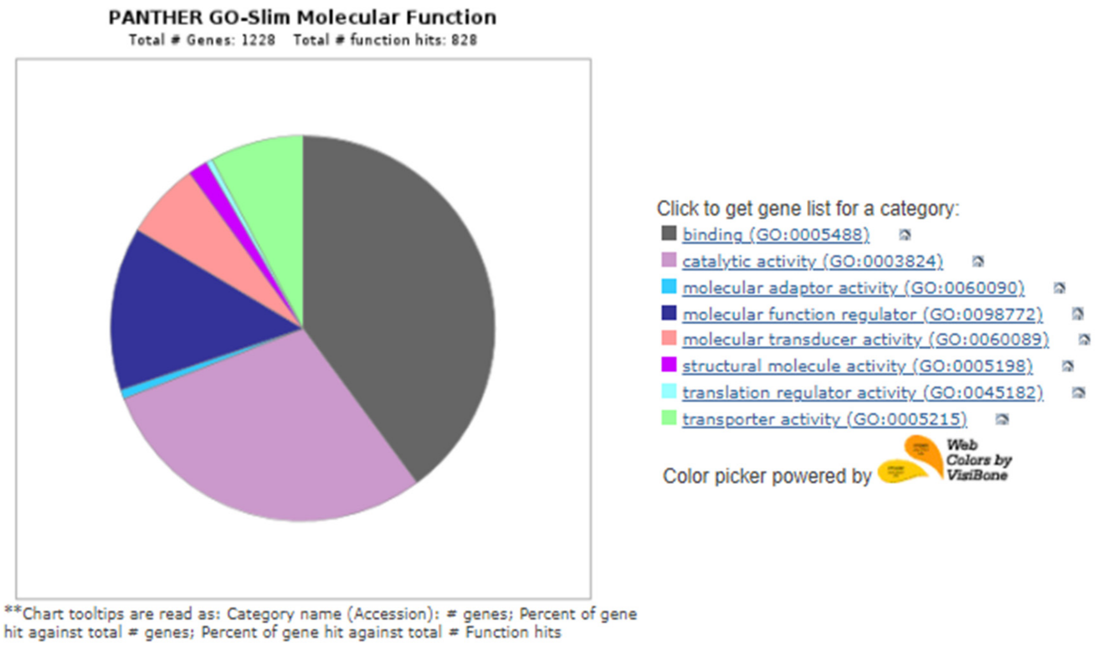

Figure S6: Results of the gene ontology analysis regarding the “molecular function” category.

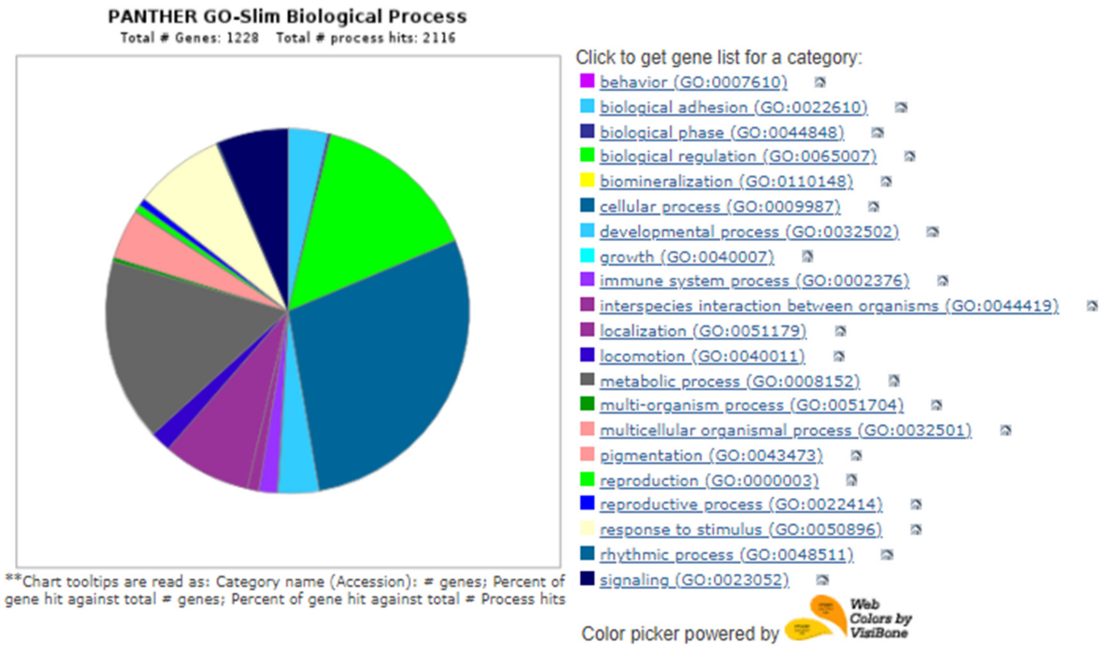

Figure S7: Results of the gene ontology analysis regarding the "biological process" category.

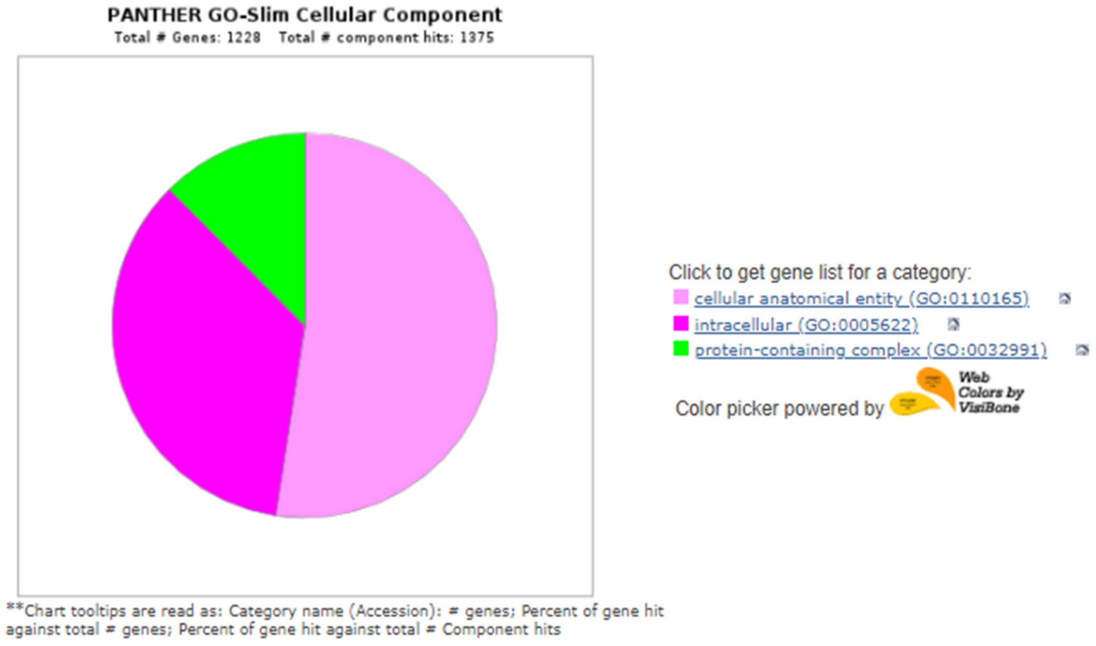

Figure S8: Results of the gene ontology analysis regarding the "cellular component" category.

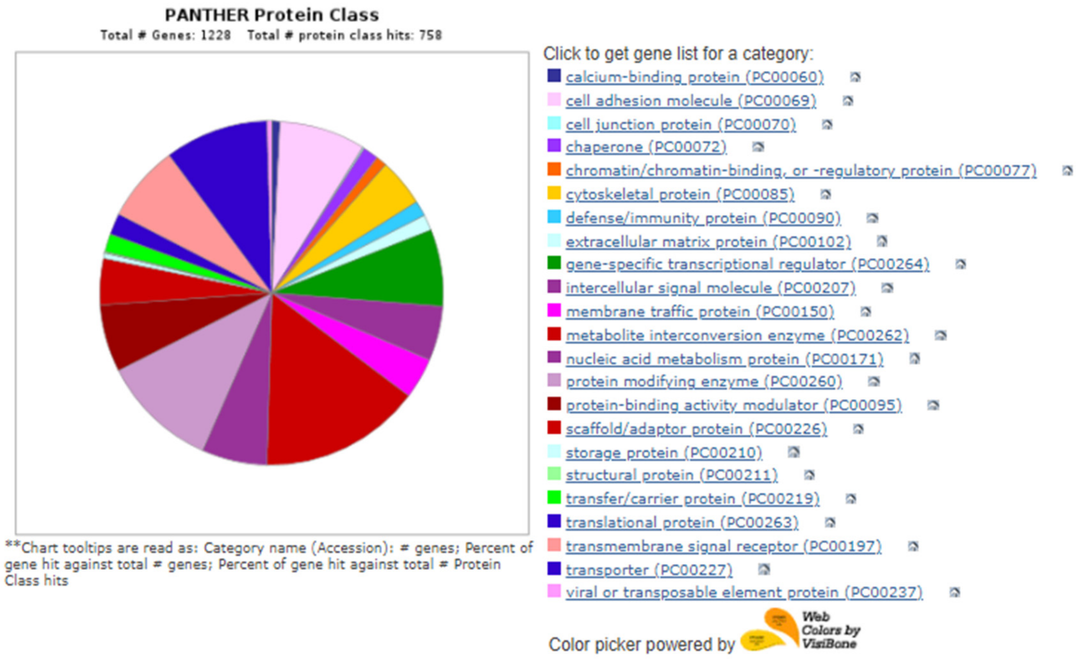

Figure S9: Results of the gene ontology analysis regarding the "protein class" category.

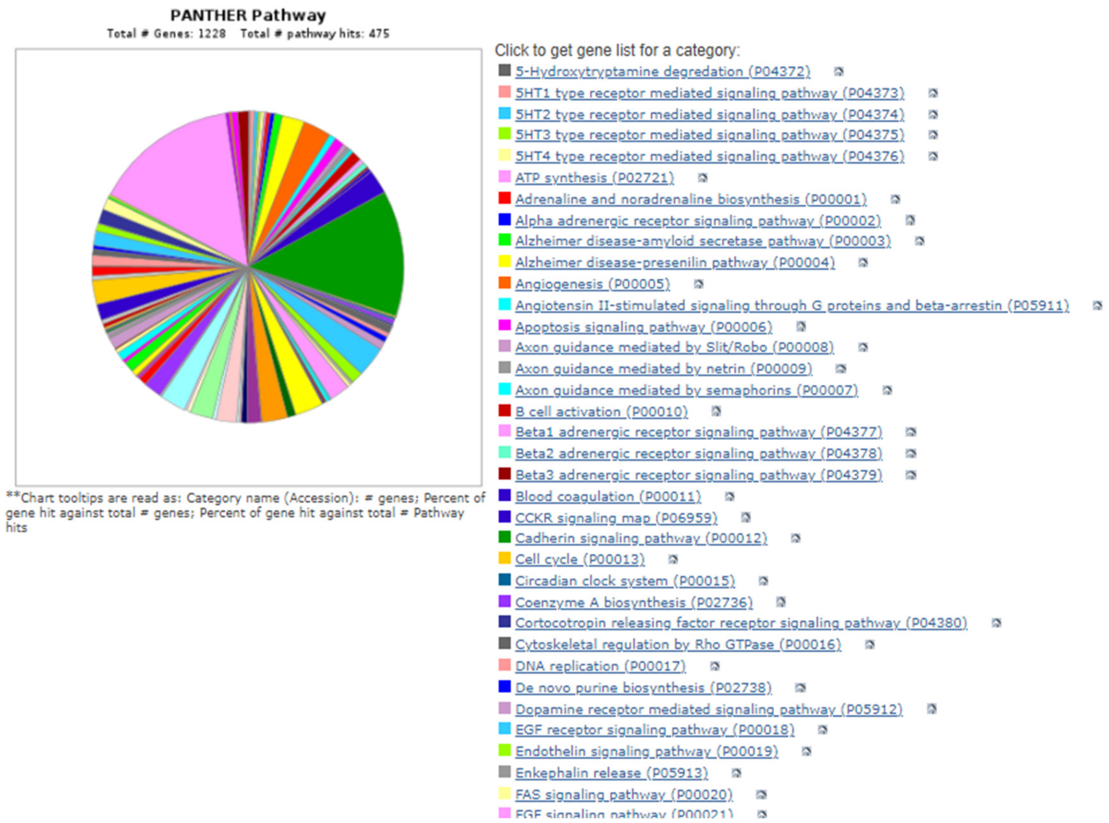

Figure S10: Results of the gene ontology analysis regarding the “pathway” category.
