# Supplementary material for: Meta-Analysis and Bioinformatics Detection of Susceptibility Genes in Diabetic Nephropathy
Source: Int J Mol Sci. 2021 Dec 21;23(1):20. doi: 10.3390/ijms23010020 (PMC8744540; doi:10.3390/ijms23010020)
Supplement: Supplementary file 1 [file ijms-23-00020-s001.zip › Supplementary Tables.pdf]

Table S1: The common genes between type 1 diabetes mellitus (T1DM) and type 1 diabetes mellitus (T2DM).

Genes common in T1DM and T2DM	*TFIP11, CCDC116, APOL2, DDT, NCF4, HIC2, SPECC1L, TTC37, CCT8L2, PCSK1, RNF185, SUSD2, CHD1, SYN3, TANGO2, GGT2, SLC25A46, CECR2, RFPL2, PRR16, ARL14EPL, RIMBP3B, NEFH, TMEM191C, FAM174A, ST8SIA4, PIWIL3, CETN3, GAS2L1, TICAM2, TEX43, FTMT, CEP120, CRYBA4, DEPDC5, ZMAT5, LIF, NUDT12, HPS4, INPP5J, ZNF70, PJA2, IGLL1, DUSP18, RTCB, RIMBP3, MEGF10, CLTCL1, SLC25A18, YWHAH, CRYBB3, CCDC188, TMED7, TEX33, EIF3D, POLR3G, SNCAIP, GAB4, FAM81B, RANBP1, PEX26, OSM, GGTLC2, SLC7A4, YTHDC2, CACNG2, PRDM6, IGLL5, USP18, HSCB, PGGT1B, SPATA9, KCTD17, MCM5, DMXL1, YPEL1, ARSK, GAL3ST1, SRRD, APOL1, SCARF2, UBE2L3, ATP6V1E1, DTWD2, TMEM121B, TPST2, ZNF74, FBXO7, HMGXB4, TBC1D10A, XKR3, SLCO6A1, PES1, ZNF608, MMP11, CABP7, SNAP29, PHAX, HORMAD2, DGCR8, GGT1, PAM, HMOX1, ERAP2, FEM1C, EFNA5, ADA2, LMNB1, RSPH14, CCDC117, C5orf63, RFPL3, MAPK1, UQCR10, GP1BB, APOL5, VPREB1, GNAZ, SF3A1, LVRN, BCR, DRICH1, RIOK2, UPB1, UFD1, ZNRF3, C1QTNF6, SFI1, AP1B1, ESS2, PVALB, STARD4, GSC2, EIF4ENIF1, CDC45, MTFP1, CABIN1, FER, CHEK2, USP41, IL17RA, FBXL17, MCC, CAMK4, TST, LYSMD3, CHCHD10, GLRX, MED15, RBFOX2, ZNF474, CASTOR1, SEC14L2, ISX, SMTN, IFT27, SEC14L6, SEZ6L, VCAN, TSLP, GPR150, CCDC157, OSBP2, LOX, GUCD1, PPIP5K2, KCNN2, DDTL, RASA1, NREP, FAM170A, C22orf39, ASPHD2, PPIC, LRRC75B, ATG12, AIFM3, TMEM232, TMEM211, C22orf15, SDF2L1, TSSK2, TXN2, GRAMD2B, COX7C, APC, CTXN3, TCN2, ZNF280A, TRIM36, RGL4, GGT5, COMT, SLC5A4, TRMT2A, RTL10, MCTP1, MEF2C, DERL3, BID, RGMB, EPB41L4A, LZTR1, ARVCF, THOC5, NR2F1, TOP3B, TIMP3, LNPEP, APOL6, RTN4R, SLC25A1, PI4KA, SMARCB1, RASD2, MYH9, HSD17B4, APOL4, RNF215, ADORA2A, GRK3, NIPSNAP1, TMEM161B, ERAP1, HIRA, TMPRSS6, GNB1L, BPIFC, SEC14L3, TXNRD2, PRR14L, POU5F2, EMID1, C22orf42, P2RX6, SGSM1, GGTLC3, TMEM191B, YDJC, WDR36, ASCC2, TBX1, SNX2, GSTT2,*

	SELENOM, MAN2A1, RFESD, ALDH7A1, TOM1, GSTT4, RAB36, BCL2L13, DRG1, SRP19, CCDC192, PRRC1, LIX1, SERPIND1, C22orf31, SEMA6A, LIMK2, MVP, PLA2G3, EDIL3, SEPTIN5, ZDHHC8, MN1, CLDN5, CDO1, FOXRED2, TUBA8, KREMEN1, MB, GSTT2B, SLC35E4, TTC28, LARGE1, REEP5, ARRDC3, KIAA1671, THAP7, LRRC74B, PRODH, COMMD10, CRYBB1, PPIL2, KLHL22, TNFAIP8, VPREB3, OR11H1, PATZ1, MICAL3, FAM172A, DCP2, GIN1, MRPL40, TMED7-TICAM2, AP3S1, ZNF280B, XBP1, CCNH, RASL10A, DGCR6, MBLAC2, SLF1, DGCR6L, POTEH, CSNK1G3, EWSR1, SRFBP1, TSSK1B, MARCHF3, HDHD5, RHOBTB3, SEC14L4, SLC5A1, PPM1F, SNRPD3, MIF, RHBDD3, SNX24, MORC2, ELL2, PRAME, CRYBB2, RIMBP3C, ADGRV1, NF2, CAST, MTMR3, DGCR2, CRKL, MYO18B, CCDC112, APOL3, IL2RB, SLCO4C1, PITPNB, PISD, CSF2RB, KIAA0825, HAPLN1, RFPL1, SLC2A11, PIK3IP1, MPST

Table S2: CytoHubba results regarding the next top 10 hub genes in T1DM and T2DM protein networks.

Official Gene Symbol	Official Full Name	Score	Cytogenetic location
**T1DM**			
PIK3R1	phosphoinositide-3-kinase regulatory subunit 1	29	5q13.1
MAPK3	mitogen-activated protein kinase 3	29	16p11.2
MRPS7	mitochondrial ribosomal protein S7	28	17q25.1
NSA2	NSA2 ribosome biogenesis factor	27	5q13.3
RPL18	ribosomal protein L18	27	19q13.33
RPL13A	ribosomal protein L13a	27	19q13.33
SNRPD3	small nuclear ribonucleoprotein D3 polypeptide	27	22q11.23
MAPK1	mitogen-activated protein kinase 1	27	22q11.22
RPL38	ribosomal protein L38	25	17q25.1
MTREX (SKIV2L2)	Mtr4 exosome RNA helicase	23	5q11.2
**T2DM**			
PPP2CA	protein phosphatase 2 catalytic subunit alpha	16	5q31.1
CFTR	CF transmembrane conductance regulator	16	7q31.2
SNRPN	small nuclear ribonucleoprotein polypeptide N	16	15q11.2
RPS14	ribosomal protein S14	15	5q33.1

PDGFRB	platelet derived growth factor receptor beta	14	5q32
COMT	catechol-O-methyltransferase	14	22q11.21
CAMK2A	calcium/calmodulin dependent protein kinase II alpha	14	5q32
IL4	interleukin 4	14	5q31.1
HSPA9	heat shock protein family A (Hsp70) member 9	14	5q31.2
UBE2D2	ubiquitin conjugating enzyme E2 D2	14	5q31.2
